# Extreme Genomic CpG Deficiency in SARS-CoV-2 and Evasion of Host Antiviral Defense

**DOI:** 10.1093/molbev/msaa094

**Published:** 2020-04-14

**Authors:** Xuhua Xia

**Affiliations:** m1 Department of Biology, University of Ottawa, Ottawa, ON, Canada; m2 Ottawa Institute of Systems Biology, University of Ottawa, Ottawa, ON, Canada

**Keywords:** SARS-CoV-2, viral evolution, canine intestine, zinc finger antiviral protein, COVID-19

## Abstract

Wild mammalian species, including bats, constitute the natural reservoir of betacoronavirus (including SARS, MERS, and the deadly SARS-CoV-2). Different hosts or host tissues provide different cellular environments, especially different antiviral and RNA modification activities that can alter RNA modification signatures observed in the viral RNA genome. The zinc finger antiviral protein (ZAP) binds specifically to CpG dinucleotides and recruits other proteins to degrade a variety of viral RNA genomes. Many mammalian RNA viruses have evolved CpG deficiency. Increasing CpG dinucleotides in these low-CpG viral genomes in the presence of ZAP consistently leads to decreased viral replication and virulence. Because ZAP exhibits tissue-specific expression, viruses infecting different tissues are expected to have different CpG signatures, suggesting a means to identify viral tissue-switching events. The author shows that SARS-CoV-2 has the most extreme CpG deficiency in all known betacoronavirus genomes. This suggests that SARS-CoV-2 may have evolved in a new host (or new host tissue) with high ZAP expression. A survey of CpG deficiency in viral genomes identified a virulent canine coronavirus (alphacoronavirus) as possessing the most extreme CpG deficiency, comparable with that observed in SARS-CoV-2. This suggests that the canine tissue infected by the canine coronavirus may provide a cellular environment strongly selecting against CpG. Thus, viral surveys focused on decreasing CpG in viral RNA genomes may provide important clues about the selective environments and viral defenses in the original hosts.

Coronaviruses (CoVs) evolve in mammalian hosts and carry genomic signatures of their host-specific environment, especially the host-specific antiviral and RNA modification activities. Many pathogenic single-stranded RNA viruses, including CoVs, exhibit strong CpG deficiency (Yap et al. 2003; Greenbaum et al. 2008, 2009; Atkinson et al. 2014; Takata et al. 2017). Two mammalian enzymes are inferred to contribute to the observed CpG deficiency. The zinc finger antiviral protein (ZAP, known as ZC3HAV1 in mammals or hZAP in human), a key component in mammalian interferon-mediated immune response, binds specifically to CpG dinucleotides in viral RNA genomes via its RNA-binding domain (Meagher et al. 2019). ZAP inhibits viral replication and mediates viral genome degradation (Takata et al. 2017; Ficarelli et al. 2019, 2020; Meagher et al. 2019). ZAP has two isoforms (ZAP-L and ZAP-S); both participate in initiating antiviral activities but only ZAP-S mediates the return to homeostasis after the antiviral response (Schwerk et al. 2019). ZAP acts against not only retroviruses, such as HIV-1 (Ficarelli et al. 2019, 2020), but also Echovirus 7 (Odon et al. 2019) and Zika virus (Trus et al. 2020), both being positive-sense single-stranded RNA viruses like CoVs. In particular, selection against CpG in viral RNA disappears in ZAP-deficient cells (Takata et al. 2017), suggesting that ZAP may be the only cellular agent targeting CpG in viral RNA genomes.

Experimental evidence is consistent with the interpretation that CpG deficiency in RNA viruses has evolved in response to these cytoplasmic CpG-specific antiviral activities. During natural evolution of HIV-1 within individual patients, viral fitness decreased with increasing CpG dinucleotides (Theys et al. 2018). Experimental increase of CpG dinucleotides in CpG-deficient viral genomes consistently leads to strong decrease in viral replication and virulence (Burns et al. 2009; Tulloch et al. 2014; Antzin-Anduetza et al. 2017; Fros et al. 2017; Wasson et al. 2017; Trus et al. 2020), prompting the proposal of vaccine-development strategies involving increasing CpG to attenuate pathogenic RNA viruses (Burns et al. 2009; Tulloch et al. 2014; Trus et al. 2020; Ficarelli et al. 2020).

Another antiviral enzyme is APOBEC3G, found in innate immune cells. APOBEC3G was originally thought specific to single-stranded DNA, such as reverse-transcribed HIV-1, but is now known to modify a variety of RNA viruses, deaminating C to U (Sharma et al. 2015, 2016, 2019). This would be effective against RNA viruses if the deaminated sites are functionally important. APOBEC3G copurifies with highly edited mRNA substrates (Sharma et al. 2016) and therefore could act on CoV genomes which are positive-sense single-stranded RNA. Although APOBEC3G is not strongly CpG-specific, it could contribute to CpG deficiency when coupled with ZAP-mediated antiviral activities targeting CpG. Modification of CpG to UpG in nonfunctional regions could reduce viral susceptibility to CpG-mediated attack by ZAP relative to viruses with unmodified CpG dinucleotides.

Both ZAP and APOBEC3G exhibit tissue-specific expression patterns in human (Fagerberg et al. 2014). Both are expressed in lungs, but ZAP is the most highly expressed where lymphocytes are the most abundant (bone marrow, lymph node, appendix, and spleen), whereas APOBEC3G is the most highly expressed in lymph node, spleen, and testis (Fagerberg et al. 2014). A severely CpG deficient virus may indicate an evolutionary history in ZAP-abundant tissues, such as strongly CpG-deficient HIV-1, infecting host T cells in lymph organs where ZAP is abundant (Fagerberg et al. 2014). The presence of such viruses indicates that they have found ways to evade ZAP-mediated cellular antiviral defense.

The differential expression of ZAP and APOBEC3G in different host or host tissues is expected to leave different genomic signatures on viral RNA genomes. We may use the conventional index of CpG deficiency (Cardon et al. 1994; Karlin et al. 1997) implemented in DAMBE (Xia 2018):

(1) $$I_{\mathrm{CpG}}=\frac{P_{\mathrm{CpG}}}{P_{C}P_{G}}.$$

The index is expected to be 1, with no deficiency or excess; <1, if deficient; and >1, if excess. The 1252 betacoronavirus (BetaCoV) full-length genomes deposited in GenBank (of which 1,127 are unique) have mean±SE value of 0.516 ± 0.0017 for *I*_CpG_, which is significantly (*P* < 0.0001) smaller than their null expectation of 1.

If a CoV infects a different host tissue with different ZAP abundance, then its RNA genome will experience different selection pressure against its CpG. This difference in cellular antiviral activity would result in differences in *I*_CpG_ during viral genomic evolution. In contrast, a CoV infecting a specific host tissue for a long time would experience the same cellular antiviral and RNA modification environment and is consequently expected to have similar and stable *I*_CpG_.

## Results

**Fig. 1:**
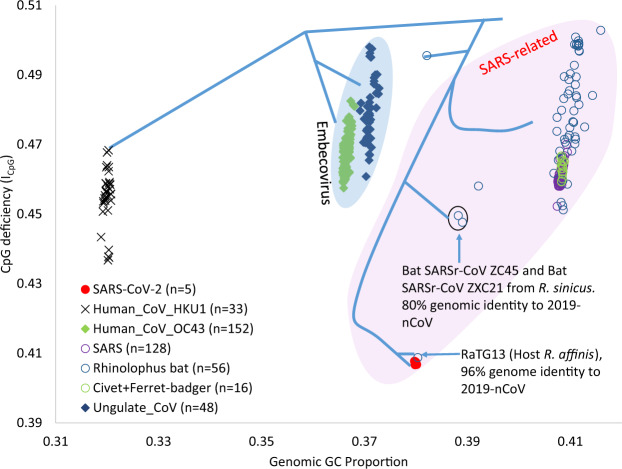
Different host species of BetaCoV have different combinations of viral genomic GC% and $I_{\mathrm{CpG}}(=P_{\mathrm{CpG}}/P_{C}P_{G})$. SARS-CoV-2 and BatCoV RaTG13 are clear outliers with extraordinarily small *I*_CpG_ values indicative of a host tissue with strong selection against CpG in the viral genome. The first three legends are viral taxonomic names. SARS: SARS CoVs, Rhinolophus bats: BetaCoVs isolated from bats in the genus *Rhinolophus*, Civet+Ferret-badger: BetaCoVs isolated from civet and ferret badger, Ungulate_CoV: BetaCoVs isolated from ungulates (including bovine and equine CoV as well as porcine hemagglutinating encephalomyelitis virus). Human CoV HKU1 and Human CoV OC43 are two members of the viral species BetaCoV1.

SARS-CoV-2 and its most closely related known relative (BatCoV RaTG13) have the lowest *I*_CpG_ among its close relatives, both being outliers in a plot of viral genomic *I*_CpG_ vs. GC% (fig. 1). Three groups of BetaCoV most closely related to SARS-CoV-2 are represented in figure 1. Group 1 consists of genomes of human CoV-HKU1 which is found only in human (Dominguez et al. 2012) and circulates among human populations without any dependence on other mammalian species as intermediate or reservoir species. Group 2 includes BetaCoV 1 genomes with two types of hosts: 1) ungulates (with bovine and equine CoV as well as porcine hemagglutinating encephalomyelitis virus); and 2) human, with CoV-OC43 being a recent derivative of bovine CoVs (Hulswit et al. 2019). Group 3 are all SARS-related CoVs from three types of hosts: 1) *Rhinolophus* bats which serve as a natural reservoir of SARS-related CoVs (Li et al. 2005; Wu, Yang, Ren, He, et al. 2016; Wu, Yang, Ren, Zhang, et al. 2016) and the new SARS-CoV-2 (Zhou et al. 2020), 2) civets (from which CoV genomes with 99.6% identity to SARS virus genomes were identified; Shi and Hu, 2008), and 3) human patients infected by SARS-CoV-2. Figure 1 shows that genomic GC% and *I*_CpG_ can differ among different viral lineages in the same host or among different hosts for the same viral lineage.

The most striking pattern in figure 1 is an isolated but dramatic shift in the lineage leading to BatCoV RaTG13 which was reported (Zhou et al. 2020) to be sampled from a bat (*Rhinolophus affinis*) in Yunnan Province in 2013 but only sequenced by Wuhan Institute of Virology after the outbreak of SARS-CoV-2 infection in late 2019. This bat CoV genome is the closest phylogenetic relative of SARS-CoV-2 (Zhou et al. 2020), sharing 96% sequence similarity. Many studies have shown an association between decreased CpG (low *I*_CpG_) in viral RNA genomes and increased virulence, not only in HIV evolving within individual patients (Theys et al. 2018) but also in experimentally CpG dinucleotide-enriched viral genomes (Burns et al. 2009; Tulloch et al. 2014; Antzin-Anduetza et al. 2017; Fros et al. 2017; Wasson et al. 2017; Trus et al. 2020). The association between decreased CpG and increased virulence in RNA viruses is mainly due to interferon-induced ZAP protein which binds to CpG dinucleotides in viral RNA genomes by its RNA-binding domain (Meagher et al. 2019), inhibits viral replication, and facilitates viral genome degradation (Takata et al. 2017; Ficarelli et al. 2019, 2020; Meagher et al. 2019). Thus, a decreased *I*_CpG_ in a viral pathogen suggests an increased threat to public health, but an increased *I*_CpG_ decreases the threat because such viral pathogens, with increased *I*_CpG_ and reduced virulence, would be akin to natural vaccines. Many viral researchers have in fact proposed vaccine development by increasing CpG in viral RNA genomes (Burns et al. 2009; Tulloch et al. 2014; Trus et al. 2020; Ficarelli et al. 2020).

In this context, it is unfortunate that BatCoV RaTG13 was not sequenced in 2013; otherwise, the downshifting in *I*_CpG_ might have served as a warning due to two highly significant implications. First, the virus likely evolved in a tissue with high ZAP expression which favors viral genomes with a low *I*_CpG_. Second and more importantly, survival of the virus indicates that it has successfully evaded ZAP-mediated antiviral defense. In other words, the virus has become stealthy and dangerous to humans.

The *I*_CpG_ value for BatCoV RaTG13 genome is 0.40875, much lower than *I*_CpG_ values observed in all other BetaCoV genomes sampled from bat species in the genus *Rhinolophus*. There are 56 BetaCoV genomes sampled from *Rhinolophus* bats inhabiting south and southeastern Asia (but mostly from central and southern China). Nature had essentially inoculated BetaCoVs into various *Rhinolophus* lineages and allowed genomic evolution to happen (supplementary fig. 1, Supplementary Material online). Although genomic *I*_CpG_ values have fluctuated in different viral lineages, only BatCoV RaTG13 has been observed to possess an extraordinarily low *I*_CpG_ (supplementary fig. 1, Supplementary Material online). This suggests that the ancestor of BatCoV RaTG13 and SARS-CoV-2 may have evolved in a mammalian tissue with high expression of ZAP and emerged with an unusually low *I*_CpG_. This mammalian tissue likely is not in *Rhinolophus* bats because low *I*_CpG_ has not been observed in other BatCoV lineages (supplementary fig. 1, Supplementary Material online). Identifying a virus with comparably low *I*_CpG_ would suggest candidate host species possessing tissues with cellular environments that select strongly against CpG in viral genomes.

**Fig. 2:**
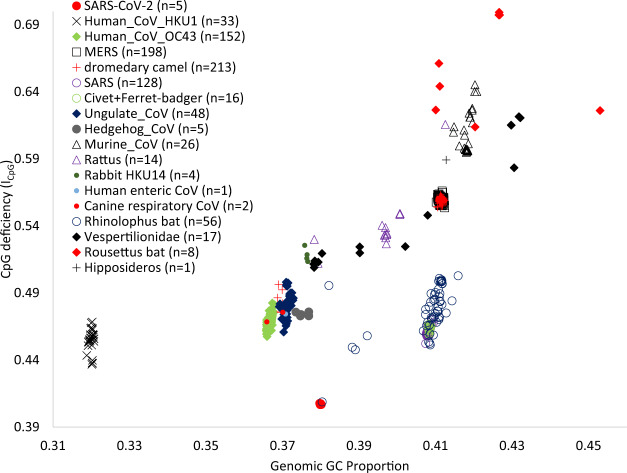
Genomic GC proportion and *I*_CpG_ for all known BetaCoVs, with a complete genome (≥27,000 nt) and host information. No BetaCoVs from their natural hosts have the genomic *I*_CpG_ and GC% combination close to SARS-CoV-2 and BatCoV RaTG13. New legends not explained in figure 1 are: MERS: MERS CoV; dromedary camel: BetaCoVs isolated from dromedary camels; Hedgehog CoV, Murine CoV, Rattus: BetaCoVs isolated from hedgehog, mouse, and rats; Rabbit HKU14: BetaCoV HKU14 strains isolated from rabbit; Human enteric CoV and Canine respiratory CoV are taxonomic names; Rhinolophus bat, Vespertilionidae, Rousettus bat, Hipposideros: BetaCoV isolated from bats in the genus *Rhinolophus*, in the family Vespertilionidae, in the genus *Rousettus*, and in the genus *Hipposideros*, respectively.

Among all BetaCoVs available in GenBank on February 3, 2020, there are 1,127 unique genomes of which 927 genomes have explicit host designations (supplementary file Betacoronavirus_CpG.xlsx, Supplementary Material online). Surprisingly, no available BetaCoV genome from diverse natural hosts has a genomic *I*_CpG_ and GC% combination close to that observed in SARS-CoV-2 and BatCoV RaTG13 (fig. 2). BetaCoV lineages parasitizing *Rhinolophus* bats overall have relatively low *I*_CpG_ values (fig. 2).

BetaCoV infecting dromedary camels offers a weak hint that camel digestive system may select more strongly against CpG in viral genomes than camel respiratory system. Camel CoVs form two clusters. One cluster overlaps completely with MERS viruses (fig. 2) that infect mammalian respiratory system (Fehr and Perlman 2015; Li 2016). The other cluster includes camel CoV HKU23 strains positioned close to bovine CoV (grouped under “Ungulate_CoV” in figs. 1 and 2), both belonging to Embecovirus and infecting mainly mammalian digestive system but also respiratory systems (Athanassious et al. 1994; Fulton et al. 2015; Ribeiro et al. 2016; Symes et al. 2018; Chae et al. 2019). Those viruses infecting camel digestive system have lower genomic *I*_CpG_ and GC% than those infecting camel respiratory system (fig. 2).

**Fig. 3:**
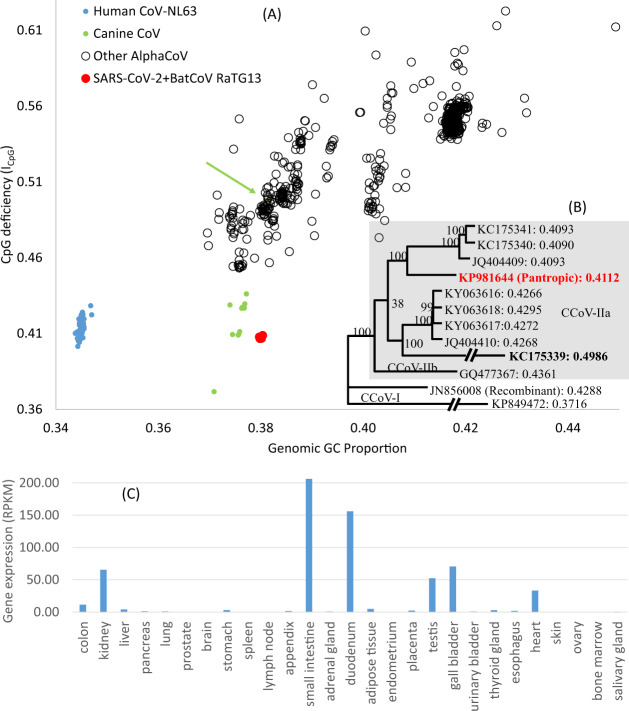
SARS-CoV-2 may have evolved in mammalian digestive tract. (*A*) Genomic GC% and *I*_CpG_ for all alphacoronaviruses with a complete genome (≥27,000 nt) and host information. Only CCoV (intestinal pathogen) genomes have GC% and *I*_CpG_ combination close to SARS-CoV-2+BatCoV RaTG13. The green arrow points to CCoV (accession KC175339 in the phylogeny) that had been propagated extensively in cell culture before sequencing. (*B*) Phylogeny from the alignment of all sequenced CCoV genomes, with leaf name in the format of (ACCN: *I*_CpG_). Genomes were aligned with MAFFT (Katoh et al. 2009) with the FFT-NS-2 option (more accurate than default). PhyML(Guindon and Gascuel 2003) with the GTR+Γ substitution model and “best” option were used to search for the best tree. (*C*) Tissue-specific gene expression of ACE2, with data from Fagerberg et al. (2014).

To search for a mammalian host with the potential to select viral lineages with low *I*_CpG_ values, I expanded the search to include all complete alphacoronavirus (AlphaCoV) genomes (supplementary file alphacoronavirus_CpG.xlsx, Supplementary Material online). All complete AlphaCoV genomes (>27,000 nt) with explicit host information are plotted in *I*_CpG_ and GC content in figure 3. Five points are worth highlighting. First, only genomes from canine coronaviruses (CCoVs), which had caused a highly contagious intestinal disease worldwide in dogs (Pratelli 2006; Le Poder 2011), have genomic *I*_CpG_ and GC% values similar to those observed in SARS-CoV-2 and BatCoV RaTG13 (fig. 3*A*). The genome (accession no. KP981644) is from the most virulent pantropic CCoV invading multiple canine organs (Buonavoglia et al. 2006; Decaro et al. 2007; Zappulli et al. 2008). It belongs to a clade with the lowest observed *I*_CpG_ values (fig. 3*B*).

Second, canids, like camels, also have CoVs infecting their respiratory system (canine respiratory CoV or CRCoV belonging to BetaCoV). There are two genomes sequenced for CRCoV (accession nos. JX860640 and KX432213). Their genomic *I*_CpG_ values are 0.4756 and 0.4684, respectively, substantially higher than those for CCoVs infecting the digestive system (fig. 3*A*). Thus, similar to the pattern observed in CoVs infecting camels, CCoVs infecting canine digestive system have *I*_CpG_ much lower than CRCoVs infecting canine respiratory system.

Third, none of the available AlphaCoV genomes from bats or other mammalian host species possess genomic *I*_CpG_ and GC% values similar to those observed in SARS-CoV-2 and BatCoV RaTG13 (fig. 3). Thus, although AlphaCoV infects a diverse array of bat lineages, these bat tissues do not seem to generate AlphaCoV strains with low *I*_CpG_ values comparable with SARS-CoV-2 and BatCoV RaTG13.

Fourth, I want to highlight one data point involving a CCoV genome represented as a green dot in figure 3 (highlighted by a green arrow in fig. 3*A*, genome accession KC175339). The CCoV has a genomic GC% of 38.17% and *I*_CpG_ of 0.4986, much higher than the rest. The virus was originally isolated from a dog but had been propagated extensively in cell culture before being sequenced (Whittaker GR, personal communication). Viruses are propagated in cells that expresses the right cellular receptor for viral entry, but do not mount an immune response to kill the virus or get killed by the virus (Benfield and Saif 1990; Banerjee et al. 2019). The consequent relaxation of selection against the virus (and against CpG in the CCoV genome) in cell culture would allow CpG in the viral RNA genome to rebound through mutation, which would explain the increased *I*_CpG_ (KC175339 in the phylogeny in fig. 3*B*). This process of regaining CpG is reminiscent of CpG-specific methylation in *Mycoplasma* species where CpG was regained when some lineages lost CpG-specific methyltransferases, with a fast-evolving lineage (*M. pneumoniae*) regaining CpG faster than a slow-evolving lineage (*M. genitalium*; Xia 2003). This rapid change in *I*_CpG_ with environmental change as shown in figure 3*B* has two important implications. First, it suggests the feasibility of tracking certain host-switching or tissue-switching events (which would be impossible if it takes hundreds of years for a virus to change *I*_CpG_). Second, many experimental studies (Burns et al. 2009; Tulloch et al. 2014; Odon et al. 2019; Trus et al. 2020; Ficarelli et al. 2020) have demonstrated attenuated virulence in RNA viruses with increasing CpG in the viral RNA genome and suggested this as an efficient means of vaccine development. The observed increase in *I*_CpG_ in the CCoV genome through cell culture propagation shows a simple way of increasing CpG by simply propagating the virus without selection against CpG dinucleotides in the viral genome.

Fifth, the cellular receptor for SARS-CoV-2 entry into the cell is angiotensin I converting enzyme 2 (ACE2; Zhou et al. 2020). ACE2 is pervasively expressed in human digestive system, at the highest levels in small intestine and duodenum (fig. 3*C*), with relatively low expression in lung (Fagerberg et al. 2014). This suggests that mammalian digestive system is likely to be infected by CoVs. This is consistent with the interpretation that the low *I*_CpG_ in SARS-CoV-2 was acquired by the ancestor of SARS-CoV-2 evolving in mammalian digestive system. The interpretation is further corroborated by a recent report that a high proportion of COVID-19 patients also suffer from digestive discomfort (Pan et al. 2020). In fact, 48.5% presented with digestive symptoms as their chief complaint.

Humans are the only other host species observed to produce CoV genomes with low-genomic *I*_CpG_ values, as shown by the cluster of human AlphaCoV NL63 genomes (fig. 3). This virus mainly not only infects the respiratory system but also causes digestive problems in 33% of the patients reporting respiratory problems (Vabret et al. 2005). In a comprehensive study of the first 12 COVID-19 patients in the United States (Midgley and The COVID-19 Investigation Team, 2020), one patient reported diarrhea as the initial symptom before developing fever and cough (Midgley and The COVID-19 Investigation Team, 2020). Stool samples from 7 out of 10 patients tested positive for SARS-CoV-2, including 3 patients with diarrhea (Midgley and The COVID-19 Investigation Team, 2020), corroborating a previous report of SARS-CoV-2 detection in stool (Holshue et al. 2020). In particular, live SARS-CoV-2 virus was isolated from stool of a COVID-19 patient (Zhang et al. 2020). In this context, it is significant that BatCoV RaTG13, as documented in its genomic sequence in GenBank (MN996532), was isolated from a fecal swab. These observations are consistent with the hypothesis that SARS-CoV-2 has evolved in mammalian intestine or tissues associated with intestine.

Figures 1 and 2 do not include all BetaCoVs from all hosts, so BetaCoVs from other mammalian species may also possess low *I*_CpG_ values. One example is viruses isolated from pangolins. Nine SARS-CoV-2-like genomes have recently been isolated and sequenced from pangolin and deposited in GISAID database (gisaid.org). The one with the highest sequence coverage (GISAID ID: EPI_ISL_410721) has an *I*_CpG_ value of 0.3929, close to the extreme low end of *I*_CpG_ values observed among available SARS-CoV-2 genomes. Thus, SARS-CoV-2, BatCoV RaTG13, and those from pangolin may either have a common ancestor with a low *I*_CpG_ or have convergently evolved low *I*_CpG_ values.

Other than ZAP and ABOBEC3G, the enigmatic DNA methyltransferase2 (Dnmt2; Okano et al. 1998a, 1998b; Dong et al. 2001), originally thought to be a Dnmt, may also contribute to viral RNA modification. However, Dnmt2 appears to methylate only small RNA (Jeltsch et al. 2017). For this reason, it may not be important in shaping *I*_CpG_ in large CoV RNA genomes, although it has been observed to relocate from the nucleus to cytoplasmic stress granules (Schaefer and Lyko 2010a, 2010b; Dev et al. 2017), where it may participate in the methylation of mRNA (Dev et al. 2017).

These observations allow formation of a hypothesis for the origin and initial transmission of SARS-CoV-2. First, the ancestor of SARS-CoV-2 and BatCoV RaTG13 infected the intestine of a mammalian species (e.g., canids or human ingesting bat meat). Second, the presumably strong selection against CpG in the viral RNA genome in canid intestine resulted in rapid evolution of the virus, with many CpG → UpG mutations leading to reduced genomic *I*_CpG_ and GC%. The licking of anal regions in canids during mating and other circumstances facilitate viral transmission from the digestive system to the respiratory system. Finally, the reduced viral genomic *I*_CpG_ allowed the virus to evade human ZAP-mediated immune response and became a severe human pathogen. Because SARS-CoV-2, TG13 and the related pangolin-derived coronaviruses all have a low-GC genome, the simplest hypothesis is that the low-GC genome was gained in their common ancestor. However, it is also possible that the viral lineage gained the low-GC genome only recently in the digestive system of a canid and spread to other species. This suggests the importance of monitoring SARS-like CoVs in feral dogs in the fight against SARS-CoV-2.

Although the specific origins of SARS-CoV-2 are of vital interest in the current world health environment, this study more broadly suggests that important evidence of viral evolution can be revealed by consideration of the interaction of host defense with viral genomes, including selective pressure exerted by host tissues on viral genome composition.

## Supplementary Material

Supplementary data are available at *Molecular Biology and Evolution* online.

## Supplementary Material

msaa094_Supplementary_Data
