# Supplementary figures and images for: Extreme Genomic CpG Deficiency in SARS-CoV-2 and Evasion of Host Antiviral Defense
Source: Mol Biol Evol. 2020 Apr 14;37(9):2699–705. doi: 10.1093/molbev/msaa094 (PMC7184484; doi:10.1093/molbev/msaa094)

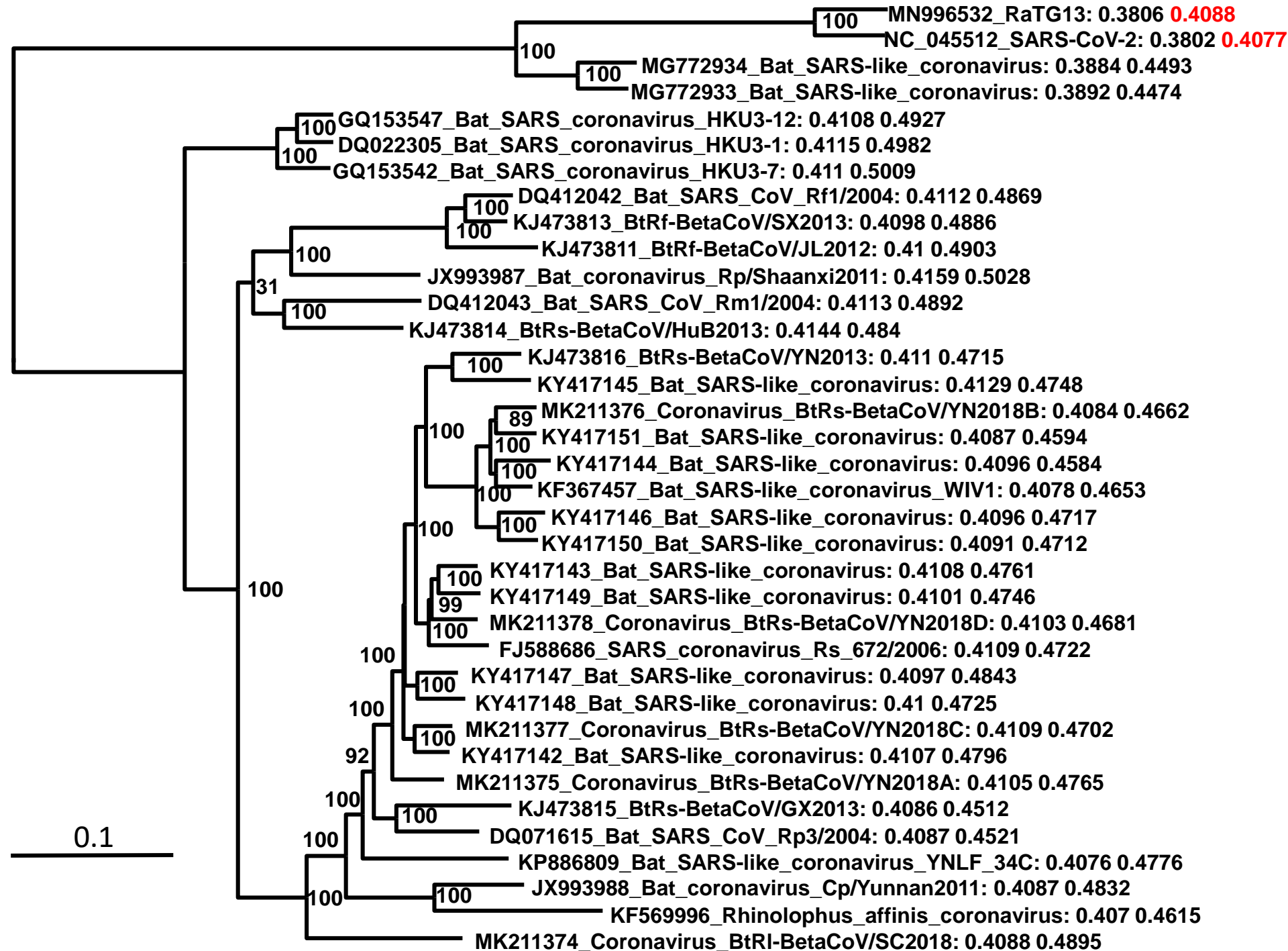

Supplement: msaa094_Supplementary_Data [file msaa094_supplementary_data.zip › msaa094-Suppl_Data/Supplemental_Fig1.pdf]
